# An Explorative Approach to Examining the Role of Ischemia and Inflammation on the Function of Autoantibodies Against G Protein–Coupled Receptors and Their Corresponding Agonists

**DOI:** 10.3390/ijms27062797

**Published:** 2026-03-19

**Authors:** Gerd Wallukat, Petra Lakatos, Kira Steinhorst, Merle Flecks, Bettina Hohberger

**Affiliations:** 1 Max Delbrück Center for Molecular Medicine, 13125 Berlin, Germany; 2 Department of Ophthalmology, University of Erlangen, Friedrich-Alexander-Universität Erlangen Nürnberg, 91054 Erlangen, Germany; petra.lakatos@uk-erlangen.de (P.L.); merle.flecks@uk-erlangen.de (M.F.); bettina.hohberger@uk-erlangen.de (B.H.)

**Keywords:** G protein–coupled receptor, autoantibody, functional autoantibody, arachidonic acid, eicosapentaenoic acid, ciliary neurotrophic factor

## Abstract

Autoantibodies (AAbs) play an important role in the development of autoimmune diseases. While many AAbs induce apoptosis of target cells, a distinct subgroup, termed functional autoantibodies (fAAbs) against G protein–coupled receptors (GPCRs), can modulate physiological receptor signaling without inducing cell death. The functional activity of GPCR-fAAbs may be influenced by various cofactors, including inflammation (e.g., inflammatory cytokine, ciliary neurotrophic factor (CNTF)) and ischemia. As ischemia triggers a substantial release of arachidonic acid (AA) from membrane phospholipids, the present study aimed to examine exploratively the influence of AA, eicosapentaenoic acid (EPA), and CNTF on the responses of spontaneously beating neonatal rat cardiomyocytes to GPCR agonists and GPCR-fAAbs. AA and EPA differentially influenced responses in cardiomyocytes induced by GPCR-fAAbs: AA altered the functional responses associated with adrenergic β_2_-fAAb, adrenergic α_1_-fAAb, angiotensin II (AT1)-fAAb, endothelin A (ETA)-fAAb and angiotensin 1–7 MAS-fAAbs. However, muscarinergic M_2_-fAAb responses remained largely unaffected. In contrast, EPA attenuated the responses to β_2_-fAAb, α_1_-fAAb, AT1-fAAb, and ETA-fAAb, while MAS-fAAb and M_2_-fAAb responses were not markedly altered. CNTF acted as a time-dependent modulator of cardiomyocyte chronotropic responses and influenced the magnitude of GPCR-mediated signaling on a cardiomyocyte bioassay. Together, these findings might suggest that lipid mediators such as AA and EPA or CNTF may modulate functional responses of cardiomyocytes associated with GPCR-fAAbs.

## 1. Introduction

It has been demonstrated that ischemia and inflammation play a pivotal role in the development of cerebral, vascular, cardiac, and other inflammation-associated diseases [1,2,3,4]. It is known that ischemic conditions are associated with the release of fatty acids and cytokines, which can modulate the response of G protein–coupled receptors (GPCRs) to agonists or functional autoantibodies (fAAbs).

Several years ago, it was demonstrated that neonatal cardiomyocytes are more sensitive to β-adrenergic stimulation when cultured under stationary conditions (i.e., ischemic conditions) than under slowly moving conditions (i.e., rocker culture with an enhanced oxygen supply) [5]. In rocker culture, the cells exhibited a typical dose–response curve to isoprenaline with a threshold concentration ranging from 0.001 to 0.01 µM and a maximal response at 1 µM. However, under stationary conditions, cultured cells were found to be more sensitive to the β-adrenergic agonist isoprenaline, with a shift in the threshold concentration to 1 pM, yet the maximal response remained constant [6]. β-adrenergic supersensitivity in rocker-cultured cardiomyocytes was observed following the application of L(+)-lactate, ω-6 fatty, AA, or 15-hydroxyeicosatetraenoic acid (15-HETE), but not D(-)-lactate [6]. In addition, melittin, an activator of phospholipase A2, induced β-adrenergic supersensitivity in neonatal cardiomyocytes, similar to that induced by L(+)-lactate or AA [6]. In the presence of L(+)-lactate or AA, rocker-cultured cardiomyocytes exhibited an atypical, elongated dose–response curve to isoprenaline with a maximal response at 1 µM and a threshold concentration ranging from 0.1 to 1 pM [6,7]. These elongated dose–response curves appear to represent a combination of two response curves. The initial segment of the curve is attributed to the β_2_ adrenoceptor, while the subsequent segment is linked to the β_1_ adrenoceptor [5]. It has been hypothesized that the β_2_ adrenoceptor is unmasked in the presence of L(+)-lactate and AA, making it accessible to hydrophilic β-agonists and β_2_-fAAb [6,8].

Previous data have shown that GPCR-fAAbs induce sustained receptor activation or in certain cases receptor inhibition, thereby disturbing the function of cardiomyocytes, including chronotropic regulation [9,10]. Interestingly, ischemic or inflammatory conditions enhance GPCR-fAAb functionality [11]. Ischemia triggers the production of lactate, known as metabolic waste, which is known to induce a substantial release of arachidonic acid (AA) from phospholipids [12]. This effect has been attributed to the activation of phospholipase A2 and can be blocked by phospholipase A2 inhibitors such as mepacrine and n-bromophenacetyl bromide. The released AA is subsequently metabolized by a lipoxygenase, resulting in the formation of 15-HETE [6]. Inflammatory processes are characterized by several factors, including a complex cytokine milieu that interferes with GPCR signaling pathways [13,14]. Currently, considerably less is known about how inflammatory cytokines affect these AAb-associated signaling pathways and how they modulate the functional impact of GPCR-fAAbs [15].Several members of the gp130 cytokine family have been implicated in cardiovascular processes, including the regulation of cardiomyocyte function under stressful conditions [16,17]. The intracellular signaling pathways activated by these cytokines are known to intersect, at least in part, with downstream cascades triggered by GPCR activation. Furthermore, altered levels of gp130-family cytokines have been reported in inflammatory and ischemia-associated cardiovascular conditions. Considering the temporal characteristics of functional GPCR-fAAb effects, we hypothesized that ciliary neurotrophic factor (CNTF) can modulate GPCR-dependent chronotropic responses.

Thus, the aim of the present study was to explore how ω-6 fatty acid (AA), ω-3 fatty acid eicosapentaenoic acid (EPA), and CNTF influence GPCR-mediated functional responses in spontaneously beating neonatal cardiomyocytes in the presence of GPCR agonists and GPCR-fAAbs.

## 2. Results

**Figure 1:**
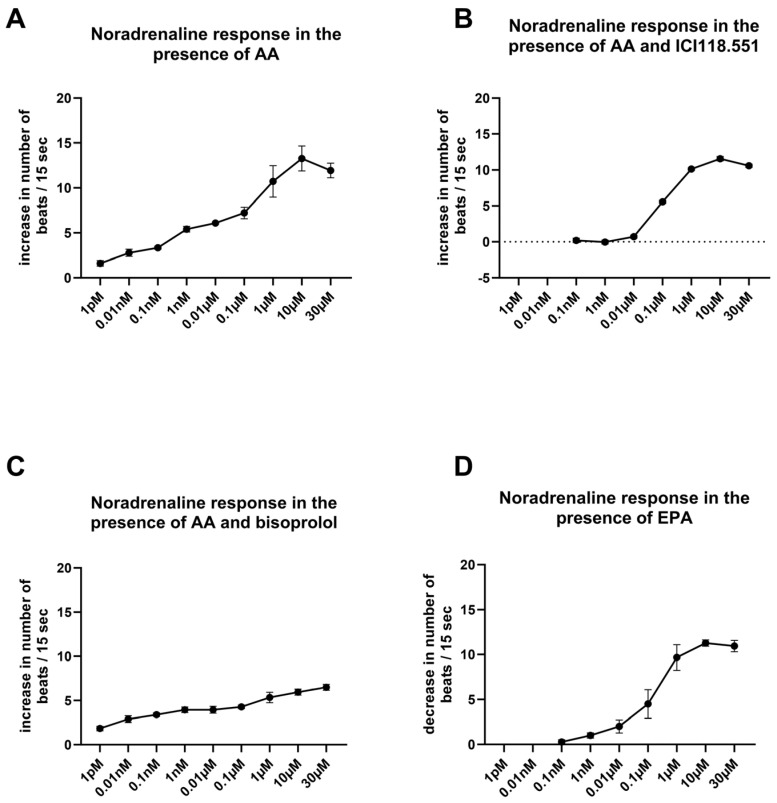
Influence of ω-6 fatty acid (AA) and eicosapentaenoic acid (EPA) on the dose response of cardiomyocytes to noradrenaline (NA). Dose–response curves for NA in spontaneously beating cardiomyocytes in the presence of AA (1 µM) (**A**), AA with the β_2_-adrenoceptor antagonist ICI118.551 (0.3 µM) (**B**), and AA with the β_1_-adrenoceptor inhibitor bisoprolol (1 µM) (**C**) and EPA (**D**). An unusually elongated dose–response curve for NA is presented with a maximal response at 10 µM and a threshold concentration at 1 pM (**A**). The overall chronotropic response reflects contributions from both β_1_ adrenoceptor and β_2_ adrenoceptor receptor–mediated signaling. The first sensitive part of the dose–response curve was strongly attenuated by the β_2_-adrenoreceptor antagonist ICI118.551 (**B**). The β_1_-adrenoceptor antagonist bisoprolol (1 µM) reduced the maximal chronotropic response and left the more sensitive portion of the curve largely unchanged (**C**). In the presence of EPA, the NA-induced chronotropic response was markedly attenuated, resulting in a dose–response profile resembling that observed in the presence of the β_2_-adrenoceptor antagonist (**D**).

Treating cardiomyocytes with AA resulted in an unusually elongated noradrenaline (NA) dose–response curve that comprised seven orders of magnitude (Figure 1A). The maximal response to NA was measured at a concentration of 10 µM, with a threshold at a concentration of 1 pM. This dose–response curve exhibited both a β_2_ adrenoreceptor–mediated response in the initial segment and a β_1_ adrenoceptor–mediated response in the subsequent segment. This biphasic dose–response behavior indicates the sequential activation of β_2_ and β_1_ adrenoceptors, with a β_2_-AR–mediated effect at the beginning. The β_2_-adrenoceptor antagonist ICI118.551 blocked the β_2_-linked part of the NA dose–response curve. Therefore, under the conditions of ICI118.551 blockade, only the β_1_ adrenoceptor–mediated effects were measurable (Figure 1B). In the presence of the inhibitor ICI118.551, the maximal NA response was observed at 10 µM with a threshold concentration of 0.01 µM. In the presence of the β_1_-adrenoceptor inhibitor bisoprolol, the NA dose–response curve was unusually elongated, yet the maximal response was halved compared to Figure 1A (Figure 1C). However, in the presence of the ω-3 fatty acid EPA (1 µM), supersensitivity to NA was completely prevented (Figure 1D). Under these conditions, the NA dose–response curve was similar to that observed after ICI118.551 administration. Unlike AA, EPA markedly attenuated the β_2_ adrenoceptor–mediated chronotropic response induced by the hydrophilic agonist noradrenaline (NA).

**Figure 2:**
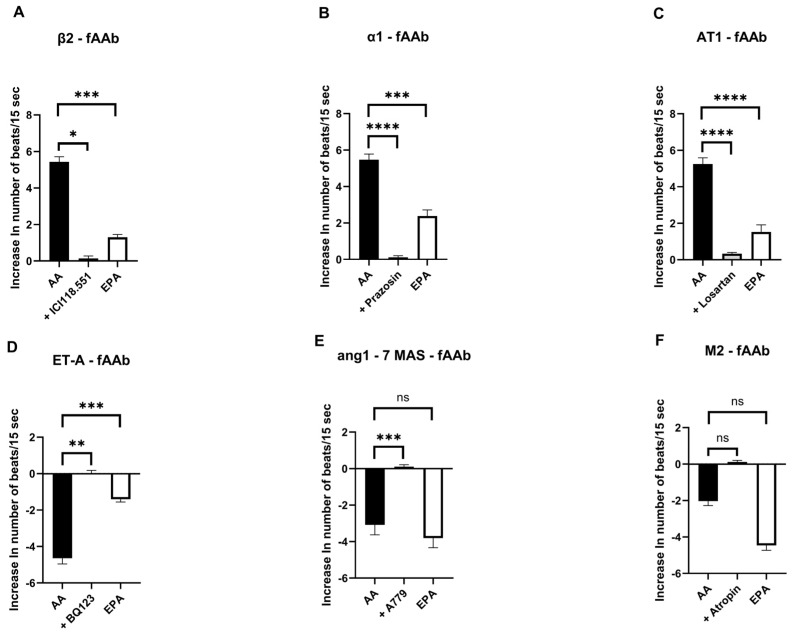
Influence of AA and EPA on cardiomyocyte responses induced by G protein–coupled receptors (GPCRs) to agonists or functional autoantibodies (fAAbs). AA modulated the responses of β_2_-fAAb (**A**), α_1_-fAAb (**B**), angiotensin II (AT1)-fAAb (**C**), endothelin A (ETA)-fAAb (**D**), and angiotensin 1–7 MAS-fAAb (**E**), whereas responses induced by muscarinic M_2_-fAAb remained largely unchanged (**F**). EPA also influenced cardiomyocyte responses to several fAAbs. In particular, EPA significantly reduced the stimulatory responses induced by fAAbs against the β_2_ adrenoceptor (**A**), α_1_ adrenoceptor (**B**), and AT1 receptor (**C**), whereas the negative chronotropic response induced by ETA-fAAbs was markedly attenuated in the presence of EPA (**D**). In contrast, EPA did not significantly alter the responses associated with angiotensin 1–7 MAS-fAAbs (**E**). Similarly, for fAAbs against the muscarinic M_2_ receptor, neither AA nor EPA resulted in statistically significant differences between conditions (**F**). Receptor-specific antagonists were used to block the respective GPCRs, resulting in attenuation or disappearance of the corresponding fAAb-induced chronotropic responses. The following inhibitors were applied: 1 µM ICI118.551 (β_2_ adrenoceptor, (**A**)), 1 µM prazosin (α_1_-adrenergic receptor, (**B**)), 1 µM losartan (AT1 receptor, (**C**)), 0.1 µM BQ123 (ETA receptor, (**D**)), 0.1 µM A779 (angiotensin 1–7 MAS receptor, (**E**)), and 1 µM 2H atropine (muscarinic M_2_ receptor, (**F**)). Δ beats/15 s values represent the mean response of 10 clusters per flask. Cardiomyocytes originated from at least three independent isolations across the study. Statistical analysis was performed as described in the Methods section. Statistical significance is indicated as ns, not significant; * *p* < 0.05, ** *p* < 0.01, *** *p* < 0.001, **** *p* < 0.0001. Data are presented as means ± SEM.

Therefore, we examined the effects of both fatty acids on chronotropic responses in cardiomyocytes induced by fAAbs directed against different GPCRs (Figure 2). On the cardiomyocyte bioassay, these fAAbs produced chronotropic responses similar to those observed after stimulation with the corresponding receptor agonists. For fAAbs directed against the β_2_ adrenoceptor, pretreatment with AA was associated with more pronounced agonist-like chronotropic responses, whereas pretreatment with EPA resulted in a markedly reduced fAAb-induced responses (Figure 2A). Similar effects were observed for fAAbs directed against other positive chronotropic GPCRs, such as the α_1_ adrenoceptor (Figure 2B) and angiotensin II (AT1) receptor (Figure 2C), as well as the endothelin 1 (ETA) receptor (Figure 2D), which acts negatively on chronotropy. The response of the cardiomyocytes to fAAbs directed against the negative chronotropic acting angiotensin 1–7 MAS receptor (Figure 2E) and M_2_-fAAb (Figure 2F) did not differ significantly between the AA and EPA treatment. Although the response appeared more pronounced under EPA and slightly attenuated under AA, these variations did not reach statistical significance. Receptor-specific inhibitors were used to block the respective GPCRs, resulting in the attenuation or neutralization of the chronotropic responses induced by the corresponding fAAbs (Figure 2A–F). Therefore, the following fAAbs were blocked: β_2_-fAAb by ICI118.551, α_1_-fAAb by 1 µM prazosin, AT1-fAAb by 1 µM losartan, ETA-fAAb by 0.1 µM BQ123, angiotensin 1–7 MAS-fAAb by 0.1 µM A779, and M_2_-fAAb by 1 µM 2H atropine.

**Figure 3:**
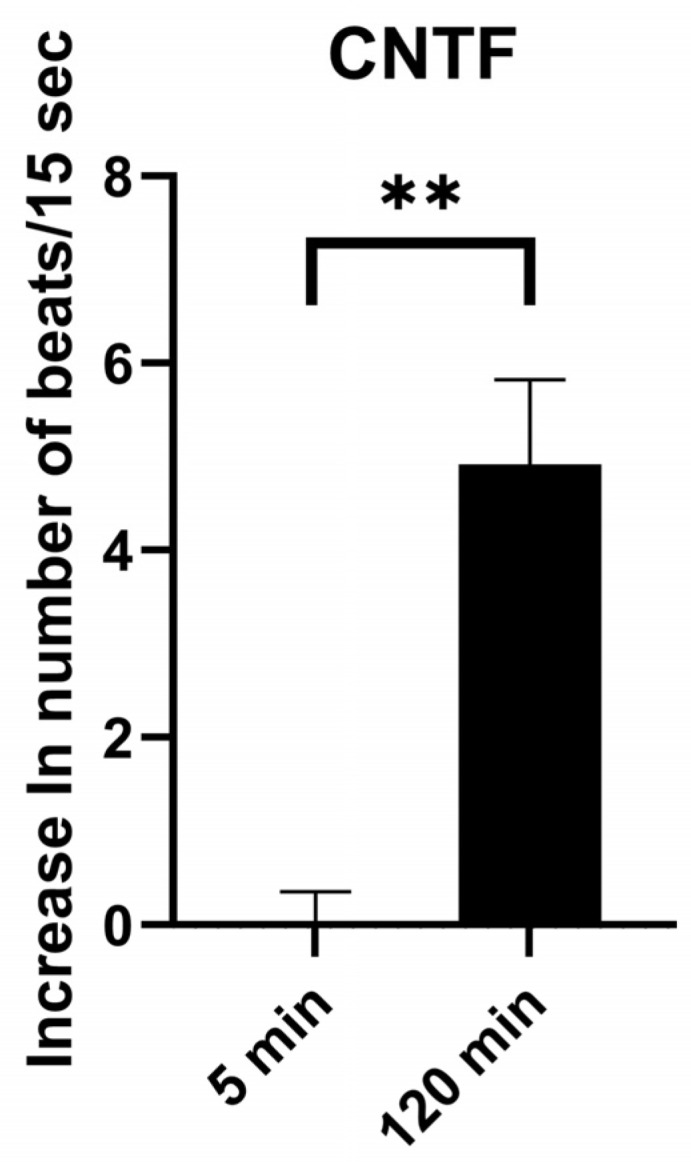
Ciliary neurotrophic factor (CNTF) induces a time-dependent increase in cardiomyocyte beating rate. No effect was observed after 5 min, whereas a moderate increase occurred after 120 min of incubation. Δ beats/15 s values represent the mean response of 10 clusters per flask. n = 4 independent experimental preparations. Normality of paired differences was assessed using the Shapiro–Wilk test and was confirmed. Therefore, paired comparisons were performed using the two-tailed paired *t*-test (complete paired observations); *p* = 0.0029. Statistical significance is indicated as ns, not significant; ** *p* < 0.01. Data are presented as means ± SEM.

**Figure 4:**
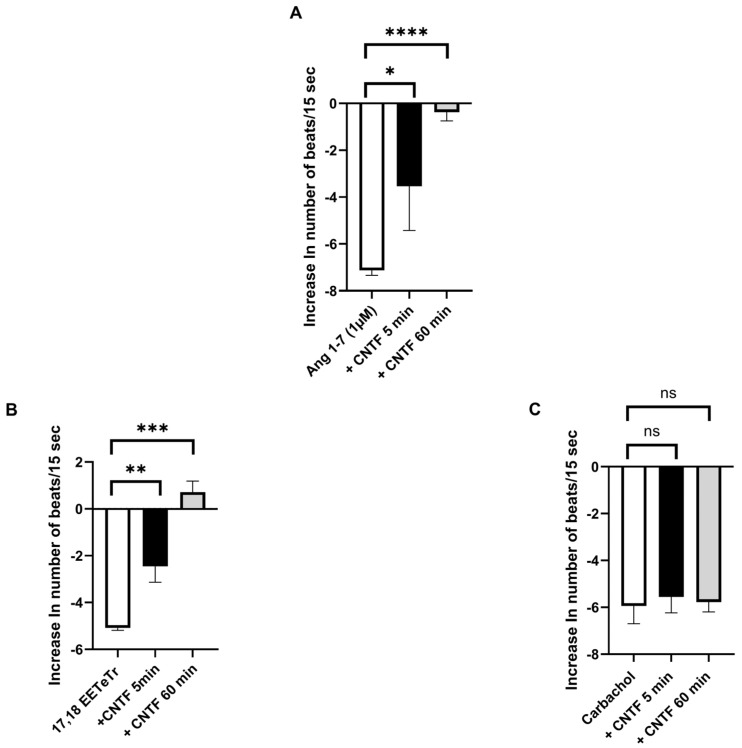
Effect of CNTF on negative chronotropic responses in cardiomyocytes. Cardiomyocyte responses to angiotensin 1–7 (Ang 1–7) (**A**), 17,18-epoxyeicosatetraenoic acid (17,18-EETeTr) (**B**), and carbachol (**C**) following CNTF incubation. CNTF altered the chronotropic responses of cardiomyocytes to Ang 1–7 and 17,18-EETeTr, whereas the muscarinic receptor–mediated response to carbachol remained largely unchanged (**A**–**C**). For both Ang 1–7 and 17,18-EETeTr, a reduction in Δ beats was already observable after 5 min of CNTF incubation and became more pronounced after 60 min. Δ beats/15 s values represent the mean response of 10 clusters per flask. Cardiomyocytes originated from at least three independent isolations across the study. Statistical analysis was performed as described in the Methods section. Statistical significance is indicated as ns, not significant; * *p* < 0.05, ** *p* < 0.01, *** *p* < 0.001, **** *p* < 0.0001. Data are presented as means ± SEM.

As shown in Figure 3, CNTF (20 pM) alone was associated with a slight increase in cardiomyocyte beating frequency over time, which occurred with delay. In a separate set of experiments, we examined the influence of CNTF on the negative chronotropic responses induced by angiotensin 1–7 (Ang 1–7, 1 µM), 17,18-epoxyeicosatetraenoic acid (17,18-EETeTr, 30 nM), and carbachol (10 µM). As illustrated in Figure 4A, the negative chronotropic response induced by Ang 1–7 was partially attenuated after 5 min of CNTF incubation and was no longer detectable after 60 min. Similar findings were observed for 17,18-EETeTr (Figure 4B). In contrast, the inhibitory response by carbachol remained largely unchanged in the presence of CNTF (Figure 4C).

**Figure 5:**
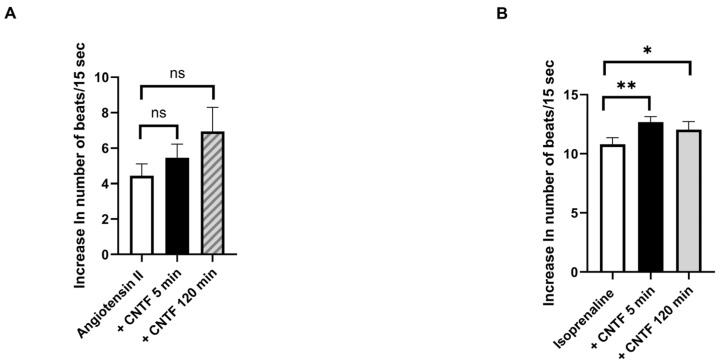
Explorative effects of CNTF on positive chronotropic response in cardiomyocytes. CNTF (20 pM) modestly enhanced the chronotropic responses induced by angiotensin II (**A**) and isoprenaline (**B**), with a modest time-dependent trend. Δ beats/15 s values represent the mean response of 10 clusters per flask. Cardiomyocytes originated from at least three independent isolations across the study. Statistical analysis was performed as described in the Methods section. Statistical significance is indicated as ns, not significant; * *p* < 0.05, ** *p* < 0.01. Data are presented as means ± SEM.

In contrast, CNTF had only a moderate effect on the positive chronotropic responses of cardiomyocytes. Following stimulation of the cells with angiotensin II (1 µM) or isoprenaline (1 µM), an increase in the beating rate of cardiomyocytes was observed. In the presence of these agonists, the addition of CNTF resulted in a further moderate increase in beating rate in both of cases (Figure 5A,B).

## 3. Discussion

GPCR-fAAbs may play a pivotal role in the development of diseases, involving the vascular system [18]. Therapeutic approaches (e.g., immunoadsorption (IA) BC007 (rovunaptabin)) have revealed a link between these GPCR-fAAbs and specific disorders. This is because an improvement in clinical symptoms was observed after their elimination (via IA) or neutralization (via rovunaptabin): IA in patients with idiopathic DCM led to the disappearance of β_1_-fAAb, accompanied by an improvement in ejection fraction and five-year survival rate [19,20]. In patients with glaucoma, IA was shown to reduce the pathological increase in intraocular pressure and eliminate β_2_-fAAb [9]. The administration of rovunaptabin led to the neutralization of the GPCR-fAAbs, followed by an improvement of fatigue in patients with post-COVID syndrome [21,22]. Previous results have indicated that the function of GPCR-fAAbs can be modified by their surrounding tissue. Inflammatory or ischemic conditions seem to be able to modify the response of cells to GPCR-fAAbs [5,6]. Furthermore, ischemia is often linked to inflammation induced by proinflammatory cytokines (e.g., cerebral ischemia) [23,24,25]. Ischemia is known to modulate the response of GPCRs to receptor-associated agonists or fAAbs, and is associated with the release of fatty acids and cytokines. Therefore, the aim of the present study was to exploratively examine the effect of selected ischemia-associated factors, namely AA, EPA, and CNTF, on the chronotropic response of cardiomyocytes to agonists and GPCR-fAAbs. The data from this study might indicate that the fatty acids AA and EPA may modulate the response of cardiomyocytes to GPCR-fAAbs differently.

Considering their effects on β_2_ adrenoceptors, the chronotropic response of cardiomyocytes to the hydrophilic agonist NA was attenuated following EPA pretreatment, in contrast to the effects of AA. EPA is particularly known to influence anti-inflammatory, vasodilatory, and ion channel–modulatory processes [26]. The responses induced by fAAbs directed against β_2_- and the α_1_ adrenoceptors, angiotensin II (AT1) receptor, and endothelin 1 (ETA) receptor showed a similar pattern to the NA-induced chronotropic response of cardiomyocytes. In contrast, responses mediated by the angiotensin 1–7 MAS receptor were affected only by AA, but not by EPA. The response induced by M_2_-fAAb remained largely unaffected by either EPA or AA. Under the experimental conditions shown, AA and EPA differed in the direction and magnitude of the observed chronotropic responses on the cardiomyocyte assay. EPA was frequently associated with reduced or altered responses compared to AA. These findings may indicate receptor-specific differences in chronotropic behavior in the presence of different polyunsaturated fatty acids, without implying baseline fAAb effects in the absence of AA or EPA.

The present results might indicate that CNTF has a time-dependent and pathway-specific effect on chronotropic responses in spontaneously beating cardiomyocytes. CNTF alone induced only a delayed and moderate increase in beating frequency. CNTF attenuated the negative chronotropic responses induced by angiotensin 1–7 and the EPA metabolite 17,18-EETeTr. However, the muscarinic receptor–associated inhibitory response remained unchanged. This pattern suggests that CNTF has differential effects on distinct chronotropic responses under the experimental conditions used. In addition to attenuating selected negative chronotropic responses, CNTF itself increased the beating rate of cardiomyocytes in a time-dependent manner and moderately enhanced the chronotropic effects induced by isoprenaline and angiotensin II. The responses to both agonists were further increased in the presence of CNTF, indicating that CNTF can modulate cardiomyocyte chronotropic responses under these experimental conditions.

The experiments were performed using a bioassay based on spontaneously beating rat cardiomyocytes. These cells respond to β-adrenergic stimulation by increasing their beating rate. However, prolonged exposure to classical agonists results in a reduced responsiveness upon restimulation, reflecting receptor desensitization. In contrast, agonistic GPCR-fAAbs induce a sustained stimulatory effect and appear to attenuate this desensitization process [6]. Such persistent receptor activation has been suggested to contribute to dysregulated cardiomyocyte function and may represent a potential pathogenic mechanism in conditions associated with GPCR-fAAbs. Previous data have shown that fAAbs interact with their corresponding receptors more extensively under ischemic conditions than under physiological conditions. This has been demonstrated for AT1-fAAbs, for example. These fAAbs stimulate the AT1 receptors of blood vessels, resulting in muscle contraction and potentially playing a role in the development of hypertension and fibrosis. Seropositivity to AT1-fAAbs has been observed in patients with hypertension, preeclampsia, and kidney diseases [27,28,29,30,31]. In an animal model (rat), contraction of the kidney arteries in response to fAAbs was observed in ischemic or inflammatory arteries, but not in healthy kidney arteries. Unlike the agonist angiotensin II, which induces a positive response in the kidney arteries, AT1-fAAbs did not recognize the receptor under healthy, non-ischemic conditions. However, under ischemic or inflammatory conditions, AT1-fAAbs stimulated AT1 receptors like the agonist angiotensin II. These effects were blocked by losartan and neutralized by peptides corresponding to the second extracellular loop of the AT1 receptor [11]. These data indicate that AT1-fAAbs recognize the receptor only when its conformation permits binding to epitopes on the second extracellular loop. Further research using an animal model of ischemic preeclampsia (the RUPP model) showed that reducing blood flow to the placenta of pregnant rats led to the development of symptoms similar to those observed in patients with preeclampsia [32]. The animals developed high blood pressure, with reduced birth weight of pups and the formation of AT1-fAAbs. These alterations were induced by proinflammatory cytokines such as IL-6 and IL-17 [33,34]. Administering a recombinant soluble IL-17 receptor (IL-17 RC) to neutralize the effects of IL-17 caused a reduction in mean arterial blood pressure (MAP) and prevented the formation of AT1-fAAbs [33,34]. However, infusing T-helper 17 (Th17) cells or IL-17 into normal pregnant rats (NP) induced AT1-fAAb formation associated with increased MAP. In some cases, these Th17 cells or IL-17–infused rats also generated fAAbs against the endothelin ETA receptor (ETA-fAAb), which has been observed in patients with severe preeclampsia and HELLP syndrome [35]. It can be hypothesized that these fAAbs could be the result of the proinflammatory cytokines, given that IL-17 levels are elevated in the first and third trimesters of women with severe preeclampsia [36,37]. Activating Treg cells with IL-10 reduced high blood pressure and diminished fAAb activity in the ischemic RUPP model. [38,39]. Thus, proinflammatory cytokines may play a role in the formation of GPCR-fAAbs, modulated by regulatory T cells (Tregs) [40]. Ischemic situations are often associated with inflammation induced by proinflammatory cytokines (e.g., cerebral ischemia) [23,24,25].

## 4. Materials and Methods

### 4.1. Immunoglobulin Preparation

The immunoglobulins (IgGs) were purified from patient serum by ammonium sulfate precipitation as described by Weir [41]. Briefly, serum was precipitated with saturated ammonium sulfate overnight at 4 °C. The samples were then centrifuged at 3376× *g*, the supernatants were discarded, and the pellets were dissolved in 0.5 L NaCl solution (154 mM NaCl, 10 mM sodium phosphate, pH 7.2). The samples were subsequently dialyzed using Membra-Cel MD44 tubing (14 kDa molecular weight cutoff; SERVA, Heidelberg, Germany) against 1 L of the same buffer at 4 °C for 4 days to remove pharmacological compounds and other low-molecular-weight biologically active substances. The dialysis buffer was changed four times. Purified IgG fractions were aliquoted and stored at −20 °C. The resulting IgG preparations had an average concentration of approximately 1 mg/mL and were later used in the cardiomyocyte bioassay at a dilution of 1:50. All materials, including solvents and chemical agents, were purchased from Merck (Darmstadt, Germany)

### 4.2. Cardiomyocyte Bioassay

The GPCR-AAb status of all patient sera had been determined prior to the experiments described here as per the cardiomyocyte bioassay protocol of Hoffmann et al. In the present manuscript, we therefore describe only the cardiomyocyte assays performed within the scope of the current study. The cardiomyocytes were isolated as described by Davideit et al. [42]. The cells were separated from the neonatal rat cardiac ventricle pieces using a 0.25% trypsin solution with constant stirring at 37 °C. The enzymatic reaction was stopped using ice-cold neonatal calf serum. The separated cells were collected and seeded into Falcon flasks at a concentration of 2.0 × 10^6^ cells/2 mL. The cardiomyocytes started rhythmically spontaneously beating after three days in culture and were used from day four in the experiments. The effects of single incubations with arachidonic acid (AA) and eicosapentaenoic acid (EPA) combined with incubation with GPCR-fAAbs were investigated. The cells were incubated in fresh culture medium 24 h prior to the experiments. All compounds were freshly prepared or diluted from the relevant stock solutions immediately prior to each experiment. For each measurement, a minimum of ten spontaneously beating cell clusters were selected. Throughout the experiments, the same cell clusters were consistently analyzed following the different treatments and incubation times to allow for paired comparisons. The cells were preincubated with AA or EPA for 30 min prior to stimulation with the respective fAAb or agonist. All pharmacological agonists and antagonists used in this study, including AA and EPA, were purchased from Sigma-Aldrich (Merck KGaA, Darmstadt, Germany).

### 4.3. Statistical Analysis

Statistical analyses were performed using GraphPad Prism (version 10.2.0; GraphPad Software, San Diego, CA, USA). Measurements were obtained from multiple flasks (experimental preparations), each representing the mean response of 10 clusters tracked across experimental conditions within the same flask. These clusters represented technical replicates within the same cardiomyocyte culture, and their responses were averaged to obtain a single value per flask. Cardiomyocytes used for these experiments were derived from at least three independent isolations during the study. Accordingly, n denotes the number of independently analyzed flasks per comparison (range: 3–9), which served as the statistical unit. Normality of paired differences was assessed using the Shapiro–Wilk test. For comparisons in which normality was confirmed, paired analyses were performed using the two-tailed paired *t*-test. Where normality was not met, the two-tailed Wilcoxon matched-pair signed-rank test was applied (complete paired observations). Data are presented as means ± SEM. A *p*-value < 0.05 was considered statistically significant. Given the limited sample size and multiple parallel comparisons, the statistical analyses should be interpreted as exploratory.

## 5. Conclusions

GPCR-fAAbs induced sustained chronotropic responses in cardiomyocytes in the bioassay. GPCR-fAAbs stimulated the cells permanently, as binding of GPCR-fAAbs might be able to disturb the binding of the agonists, thus preventing desensitization of the receptors. In the present study, AA, EPA, and CNTF modulated the effects of several GPCR-fAAbs on the beating rate of cardiomyocytes in an in vitro experimental approach. These findings might suggest that the chronotropic responses induced by GPCR-fAAbs and receptor agonists can be influenced by specific modulating factors. As negative chronotropic responses were attenuated in the presence of CNTF and positive chronotropic responses were enhanced in the presence of AA or attenuated in the presence of EPA, these findings are consistent with previous observations suggesting that GPCR-fAAb–associated effects may vary under ischemic or inflammatory conditions. The observations reported here are based on functional chronotropic responses in the cardiomyocyte bioassay and do not directly address receptor binding or downstream signaling mechanisms. Thus, further studies are necessary to combine this experimental approach with receptor-binding or -signaling analyses. It should also be noted that the IgG samples analyzed in this exploratory study were derived from previously characterized patient cohorts, and therefore a full donor-level clinical characterization was not performed within the scope of the present research.

## Data Availability

The original contributions presented in this study are included in the article. Further inquiries can be directed to the corresponding author.

## Abbreviations

The following abbreviations are used in this manuscript.

fAAbs	functional autoantibodies
GPCR	G protein–coupled receptor
AA	arachidonic acid
EPA	eicosapentaenoic acid
CNTF	ciliary neurotrophic factor
MMP	matrix metalloprotease
NA	noradrenaline
15-HETE	15-hydroxyeicosatetraenoic acid
α-adrenergic-R	α-adrenergic receptor
AT1-R	angiotensin II type 1 receptor
ETA-R	endothelin A receptor
Ang 1–7 MAS-R	Ang 1–7 MAS receptor
muscarinic M_2_-R	muscarinic M_2_ receptor
17,18-EETeTr	17,18-epoxyeicosatetraenoic acid
GRP30	G protein–coupled estrogen receptor
